# Classification complexity in myoelectric pattern recognition

**DOI:** 10.1186/s12984-017-0283-5

**Published:** 2017-07-10

**Authors:** Niclas Nilsson, Bo Håkansson, Max Ortiz-Catalan

**Affiliations:** 1 Department of Electrical Engineering, Chalmers University of Technology, Gothenburg, Sweden; 2 Integrum AB, Mölndal, Sweden

**Keywords:** Classification complexity, Myoelectric pattern recognition, Electromyography, Prosthesis control

## Abstract

**Background:**

Limb prosthetics, exoskeletons, and neurorehabilitation devices can be intuitively controlled using myoelectric pattern recognition (MPR) to decode the subject’s intended movement. In conventional MPR, descriptive electromyography (EMG) features representing the intended movement are fed into a classification algorithm. The separability of the different movements in the feature space significantly affects the classification complexity. Classification complexity estimating algorithms (CCEAs) were studied in this work in order to improve feature selection, predict MPR performance, and inform on faulty data acquisition.

**Methods:**

CCEAs such as nearest neighbor separability (NNS), purity, repeatability index (RI), and separability index (SI) were evaluated based on their correlation with classification accuracy, as well as on their suitability to produce highly performing EMG feature sets. SI was evaluated using Mahalanobis distance, Bhattacharyya distance, Hellinger distance, Kullback–Leibler divergence, and a modified version of Mahalanobis distance. Three commonly used classifiers in MPR were used to compute classification accuracy (linear discriminant analysis (LDA), multi-layer perceptron (MLP), and support vector machine (SVM)). The algorithms and analytic graphical user interfaces produced in this work are freely available in BioPatRec.

**Results:**

NNS and SI were found to be highly correlated with classification accuracy (correlations up to 0.98 for both algorithms) and capable of yielding highly descriptive feature sets. Additionally, the experiments revealed how the level of correlation between the inputs of the classifiers influences classification accuracy, and emphasizes the classifiers’ sensitivity to such redundancy.

**Conclusions:**

This study deepens the understanding of the classification complexity in prediction of motor volition based on myoelectric information. It also provides researchers with tools to analyze myoelectric recordings in order to improve classification performance.

## Background

Decoding of motor volition via myoelectric pattern recognition (MPR) has many clinical applications such as prosthetic control [1], phantom limb pain treatment [2], and rehabilitation after stroke [3]. Research on MPR has focused on classifiers [4], pre-processing algorithms [5], and electromyography (EMG) acquisition [6], among other factors that influence the classification outcome. Reaz et al. studied different attributes of EMG signals, such as signal-to-noise ratio, that decrease the complexity of MPR [7]. However, limited studies have been conducted on the complexity of the classification task itself. Information on complexity prior to classification can inform on specific conflicting classes and flawed data acquisition. Understanding of classification complexity can also be used to select optimal features and evaluate trade-offs between the amount of classes and their separability.

Most MPR algorithms use EMG features extracted from overlapping time windows as the classifier input. Therefore, the resulting classification accuracy is dependent on the features used to describe the EMG signals. The performance of a variety of such features, and feature selection algorithms, have been studied previously [8, 9]. Two feature selecting algorithms, namely minimum redundancy and maximum relevance [10], and Markov random fields [11], were applied to an electrode array by Liu et al. [12], who used Kullback–Leibler divergence and feature scatter to rate the relevance and redundancy of features. The features were then ranked and selected into sets according to these ratings. Similarly, Bunderson et al. defined three data quality indices – namely, repeatability index (RI), mean semi-principal axis, and separability index (SI) – to evaluate the changes in data quality over repeated recordings of EMG [13]. Classification complexity estimation was not investigated in the aforementioned studies, but algorithms intended to quantify attributes relevant to the complexity of pattern recognition tasks were introduced.

Classification complexity has been studied outside the field of MPR. Singh suggested two nonparametric multiresolution complexity measures: nearest neighbor separability (NNS) and purity [14]. These complexity measures were compared with common statistical similarity measures, such as Kullback–Leibler divergence, Bhattacharyya distance, and Mahalanobis distance, and were found to yield a higher correlation with classification accuracy. These classification complexity estimating algorithms (CCEAs), along with Hellinger distance, were investigated in the present study with a focus on their relevance for MPR.

In the present study, CCEAs were evaluated based on their correlation with offline classification accuracy and real-time classification performance. Consequently, different attributes were revealed about the CCEAs, classification algorithms, and features descriptiveness. One such attributes – channel correlation dependency – was investigated further. The CCEAs that were found to yield high correlation with classification accuracy (NNS and SI) were then used for feature selection and benchmarked against features sets found in the literature.

The result of these experiments provided evidence of the suitability of CCEAs to predict MPR performance. The algorithms used in this work were implemented and made freely available in BioPatRec, an open-source platform for development and benchmarking of algorithms used in advanced myoelectric control [15, 16].

## Methods

### Data sets

Two data sets were used in this study and both were recorded on healthy subjects. The first set contained individual movements (*IM* data): 20 subjects, four EMG channels, 14 bits Analog to Digital Conversion (ADC), and 11 classes (hand open/close, wrist flexion/extension, pro/supination, side grip, fine grip, agree or thumb up, pointer or index extension, and rest or no movement) [15]. The second set contained individual and simultaneous movements (*SM data*): 17 subjects, eight EMG channels, 16 bits ADC, and 27 classes (hand open/close, wrist flexion/extension, pro/supination, and all their possible combinations) [17]. Disposable Ag/AgCl (Ø = 1 cm) electrodes in a bipolar configuration (2 cm inter-electrode distance) were used in both sets. The bipoles were evenly spaced around the most proximal third of the forearm, with the first channel placed along the extensor carpi ulnaris. Subjects were seated comfortably with their elbow flexed at 90 degrees and forearm supported, leaving only the hand to move freely. The data sets, along with details on demographics and acquisition hardware, are available online as part of BioPatRec [16]. Table 1 summarizes these data sets.

**Table 1:** Summary of data sets

Reference	Movements	Subjects	Channels	ADC (bits)	Classes
*IM data*	Individual	20	4	14	11
*SM data*	Simultaneous	17	8	16	27

*Summary of the data sets used in the experiments of this study. The reference column contains the name used when referring to that data set throughout the report*

### Signal acquisition, pre-processing and feature extraction

BioPatRec recording routines guided the subjects to perform each movement three times with resting periods in between. The instructed contraction time, as well as the resting time, was 3 s. The initial and final 15% of each contraction was discarded as this normally corresponds to delayed response and anticipatory relaxation by the subject, while the remaining central 70% still preserves portions of the dynamic contraction [15].

Time windows of 200 ms were extracted from the concatenated contraction data using 50 ms time increment. Features were then extracted from each time window and distributed in sets used for training (40%), validation (20%), and testing (40%) of the classifiers. The testing sets were never seen by the classifier during training or validation. A 10-fold cross-validation was performed by randomizing the feature vectors between the three sets before training and testing.

The following EMG signal features were used as implemented in BioPatRec [15, 16, 18]. In the time domain: mean absolute value (tmabs), standard deviation (tstd), variance (tvar), waveform length (twl), RMS (trms), zero-crossing (tzc), slope sign changes (tslpch), power (tpwr), difference abs. Mean (tdam), max fractal length (tmfl), fractal dimension Higuchi (tfdh), fractal dimension (tfd), cardinality (tcard), and rough entropy (tren). In the frequency domain: waveform length (fwl), mean (fmn) and median (fmd). Feature vectors were constructed by sets of these features extracted from all channels, as commonly done in MPR and implemented in BioPatRec (for a detailed explanation see reference [15]).

### Classification complexity estimating algorithms

The classification complexity estimating algorithms (CCEAs) were designed to return classification complexity estimates (CCEs) for each movement separately (*individual result*), and averaged over all movements (*average results*). *Individual results* provide information that facilitates the choice of movements to be included in a given MPR problem by distinguishing conflicting classes. *Average result* considers the complete feature space, including all movements, and can therefore be used to evaluate and compare feature sets used to build the feature space. The CCEAs used are outlined below.

#### Separability index

**Fig. 1:**
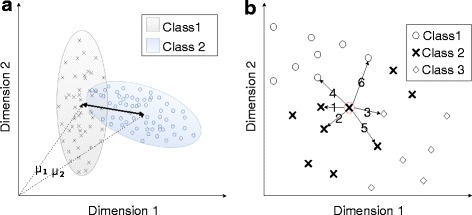
Illustration of a two-dimensional feature space. *Inset* **a** shows the distance between two classes in a two-dimensional features space. The ellipses representing the classes are constructed according to the covariance of the two-dimensional data. The figure emphasizes the overlap of classes, which is a big challenge in pattern recognition. *Inset* **b** shows the six nearest neighbors of the marked target data point. NNS is based on the fraction of the neighbors from the same class as the target point

Separability index (SI) was implemented as introduced by Bunderson et al.; that is, the average of the distances between all movements and their most conflicting neighbor [13]. Figure 1a illustrates the distance and conflict between two classes in an exemplary two-dimensional feature space.

The aforementioned distance was defined by Bunderson et al. to be half the *Mahalanobis distance*, resulting in the following equation:

$$ SI=\sum_{i=1}^K\left(\underset{j=1,\dots, i-1, i+1,\dots, K}{\mathit{\min}}\frac{1}{2}\sqrt{{\left({\mu}_i-{\mu}_j\right)}^T{S}_i^{-1}\left({\mu}_i-{\mu}_j\right)}\right) $$

where *K* is the number of classes or movements, and μ_x_ and *S*_*x*_ are mean vectors and covariance matrices for class *x,* respectively.

This definition only considers the covariance of the target movement (*S*_*i*_), and not that of the comparing movement (that is, *S*_*j*_). We considered this particular formulation as a potential limitation, so we introduced additional *distance definition*s. The *distance definitions* were used under the assumption of normality as *Mahalanobis distance* was defined under the same assumption [19]. The introduced *distance definitions* are described in Table 2 **.**

**Table 2:** Distance definitions for SI

Distance definition	Description
Mahalanobis distance	*Mahalanobis distance* was designed to measure the distance between a distribution and a single point [19]. Half the *Mahalanobis distance* will be the value referred to as *Mahalanobis distance* hereafter because that is how it was originally used in SI [13]. *Mahalanobis distance* for multivariate normal distributions is defined as: $\frac{D_M}{2}=\frac{1}{2}\sqrt{{\left({\mu}_1-{\mu}_2\right)}^T{S}_1^{-1}\left({\mu}_1-{\mu}_2\right)}$
Bhattacharyya Distance	*Bhattacharyya distance* is a measurement of statistical similarity between two distributions based on the *Bhattacharyya Coefficient* (BC) [24]. Unlike *Mahalanobis distance*, *Bhattacharyya distance* takes both the distance and similarity in covariance between the distributions into account. In this study, the square root of *Bhattacharyya distance* was used to equate the formulation of *Mahalanobis distance* and facilitate comparison. *Bhattacharyya coefficient* for the continuous probability distributions p and q is defined as: $BC=\int \sqrt{p(x) q(x)} dx$ *Bhattacharyya distance* as function of *Bhattacharyya coefficient:* $\sqrt{D_B}=\sqrt{-\frac{1}{2} \ln (BC)}$ *Bhattacharyya distance* for multivariate normal distributions (square root) [25]: $\sqrt{D_B}=\sqrt{\frac{1}{8}{\left({\mu}_1-{\mu}_2\right)}^T{S}^{-1}\left({\mu}_1-{\mu}_2\right)-\frac{1}{2} \ln \left(\frac{detS}{\sqrt{\mathit{\det}{S}_1\mathit{\det}{S}_2}}\right)}$
Kullback–Leibler divergence	*Kullback–Leibler divergence* is a well-known statistical similarity measure that is typically used to determine whether an observed distribution, *Q*, is a sample of a true distribution, *P* [26]. *Kullback–Leibler divergence* for multivariate normal distributions is defined as [25]: ${D}_{KL}=\frac{1}{2}\left( tr\left({S}_1^{-1}{S}_2\right)+{\left({\mu}_1-{\mu}_2\right)}^T{S}_1^{-1}\left({\mu}_1-{\mu}_2\right)- k+\mathit{\ln}\left(\frac{\mathit{\det}{S}_1}{\mathit{\det}{S}_2}\right)\right)$
Hellinger distance	*Hellinger distance* is related to *Bhattacharyya distance* as it is also based on the *Bhattacharyya coefficient* [27]. The square of the *Hellinger distance* was used in this study to avoid complex numbers appearing where the assumption of normality fails, and this value is referred to here as the *Hellinger distance*. *Hellinger distance* as a function of the *Bhattacharyya coefficient* is defined as: ${D}_H^2=1- BC$ *Hellinger distance* for multivariate normal distributions: ${D}_H^2=1-{\frac{{\left({detS}_1\right)}^{\frac{1}{4}}{\left({detS}_2\right)}^{\frac{1}{4}}}{{\left({detS}_1\right)}^{\frac{1}{2}}}}^{\ast}\mathit{\exp}\left\{-\frac{1}{8}{\left({\mu}_1-{\mu}_2\right)}^T{S}^{-1}\left({\mu}_1-{\mu}_2\right)\right\}$
Modified Mahalanobis	This measure of statistical similarity is equal to the aforementioned *Mahalanobis distance*, except that it takes into account the covariance matrix of both distributions being compared. The algorithm is related to *Bhattacharyya distance,* but is only focused on the distance between the distributions. This CCEA is referred to here as *modified Mahalanobis* and is defined for multivariate normal distributions as: $\frac{D_{MM}}{2}=\frac{1}{2}\sqrt{{\left({\mu}_1-{\mu}_2\right)}^T{S}^{-1}\left({\mu}_1-{\mu}_2\right)}$
Explanations	All equations above index 1 and 2 are appointed the considered movement and the compared movement, respectively, and $S=\frac{S_1+{S}_2}{2}$

*Table of distance definitions used to compute SI, including their names, definitions, and how the were implemented in the present study*

#### Nearest neighbor Separability

Nearest neighbor separability (NNS) was inspired by the algorithm with the same name defined by Singh [14]. It is based on the dominance of nearest neighbors, in feature space, belonging to the same class (movement) as a target data point. The contributions of the nearest neighbors are weighted by their proximity to the target point and the result is normalized to be a value between 0 and 1. Let

$$ b\left({p}_t,{p}_i\right)=\left\{\begin{array}{c}1,\\ {}0,\end{array}\right.\begin{array}{c} if\ {p}_t,{p}_i\in C\\ {}\kern2em  if\ {p}_t\in C,{p}_i\notin C\end{array} $$

Where *p*_*t.*_ is the target point, *p*_*i*_ is *p*_*t.*_:s *i-th* nearest neighbor and *C* is a class. The aforementioned dominance is then defined as:

$$ {d}_t={\left(\sum_{i=1}^k\frac{1}{i}\right)}^{-1}\sum_{i=1}^k\frac{b\left({p}_t,{p}_i\right)}{i} $$

A target point and its six nearest neighbors are illustrated in Fig. 1b.

The end result is the average dominance:

$$ NNS=\frac{1}{N}\sum_{i=1}^N{d}_i $$

Where *N* is the total number of samples*.*

Unless stated otherwise, the parameter *k* is set to 120, which is the maximum number of nearest neighbors from the same class for the data sets of this study.

#### Purity

Purity was computed by dividing the feature hyperspace into smaller hyper cuboids called cells [14]. The cells were rated individually and high dominance of one class in one cell meant high purity for that cell. The final purity of a data set was the average over all cells and different cell resolutions.

#### Repeatability index

The repeatability index (RI) measures how much individual classes varies between different occurrences using *Mahalanobis distance* [13]. The three repetitions during the recording session were the occurrences that were evaluated. The end result is the average *Mahalanobis distance* between the first repetition and the following ones for all movements.

### Classifiers and topologies

Three common classifiers for MPR were used in this study: linear discriminant analysis (LDA), multi-layer perceptron (MLP), and support vector machine (SVM). A quadratic kernel function was used for SVM. The classifiers were utilized as implemented in BioPatRec [15] (code available online [16]), where LDA and SVM were implemented using Matlab’s statistical toolbox.

MLP and SVM are inherently capable of simultaneous classification when provided with the feature vectors of mixed (simultaneous) outputs, hereafter referred as “MIX” output configurations; that is, there is one output for every individual movement and combinations of movements produce the corresponding mix of outputs to be turned on. LDA’s output is computed by majority voting, which means it cannot produce simultaneous classification by creating a mixed output. However, classifiers like LDA can still be used for simultaneous classification using the *label power set* strategy, where the classifier is constructed having the same number of outputs as the total number of classes. This configuration is referred to here as “all movements as individual” (AMI). Ortiz-Catalan et al. showed that AMI could also favor classifiers capable of mixed outputs [17]; therefore, MLP and SVM were evaluated in both MIX and AMI configurations for simultaneous predictions. In addition, LDA was also used in the One-Vs-One topology (OVO), as this has been shown to improve classification accuracy for individual movements [17, 20].

### Evaluation and comparison

In order to evaluate the correlation between Classification Complexity Estimates (CCEs) and classification accuracy, all features were used individually to classify all movements from each subject in both data sets, which provided a wide range of classification accuracies and their related CCEs. Correlations were then calculated considering the classification of each movements individually (*individual results*), or the average over all movements (*average results*).

The CCEAs were further used to select one set of two, three, and four features. CCEs were calculated for all possible combinations of features and the three sets – one for every number of features – predicting the highest accuracy were selected. The selected sets are referred hereafter as the *best sets* and were obtained using the *IM data* set.

Ortiz-Catalan et al. used a genetic algorithm to find optimal feature sets of two, three, and four features based on classification performance [8]. Their proposed sets of two and three features were used as benchmarking sets in this study, along with the commonly used four-feature set proposed by Hudgins et al. [21]. These sets are referred in this study as *reference sets*:

- **Ref 2F:** tstd, trms [8]
- **Ref 3F:** tstd, fwl, fmd [8]
- **Ref 4F:** tmabs, twl, tslpch, tzc [21]

The *best* and *reference sets* of equal number of features were compared to each other based on the resulting classification accuracy, as given by the three different classifiers. Classification accuracy corresponds to offline computations unless otherwise stated. Real-time testing was done using the *Motion Tests* as implemented in BioPatRec [15, 22]. CCEAs’ proficiency at predicting real-time performance was evaluated by their correlation with the *completion time* obtained from *motion tests*, which is the time from the first prediction not equal to *rest* until 20 correct predictions are achieved. Similar to offline computations, one prediction was the classification of one 200 ms time window, and new predictions were produced every 50 ms (time increment). The subject was instructed to hold the requested movement until 20 correct predictions were achieved. If the number of correct predictions was less than 20 after 5 s, the *completion time* was set to 5 s. The real-time results were obtained from *IM data* set and related *Motion Test*s [22].

Wilcoxon signed-rank test (*p* < = 0.05) was used to evaluate statistical significant differences. Correlations were calculated using Spearman’s rho, since there was no clear linearity in the dependencies between accuracy and CCE.

## Results

### Separability index (SI)

**Fig. 2:**
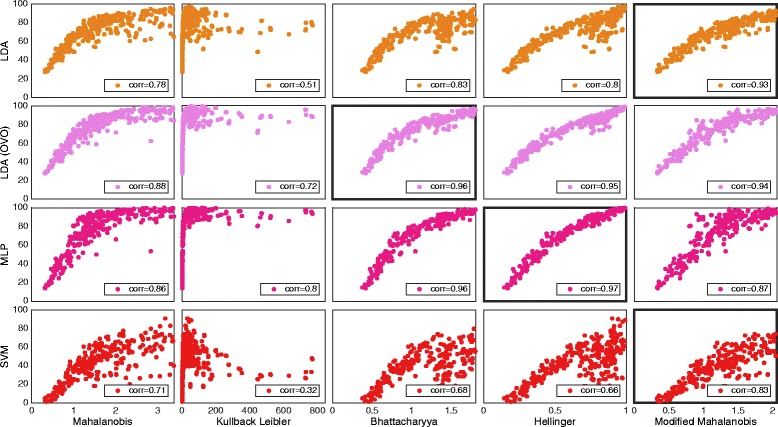
The distribution of distance definitions and classifiers data using individual movement (IM set). Plot matrix where the insets show classification accuracy plotted against the SI for the individual movements data set. One marker represents the average over all movements for one subject and one feature. The classifiers are grouped in rows and the distance definitions for the SI are grouped in columns. Classifiers were used in the conventional “single” topology, apart from LDA, which was used in “single” and “one vs. one” (OVO). All correlations were found statistically significant at *p* < 0.01. Classifiers and distance definitions are stated at the left side and the bottom of the plot matrix, respectively. The highest correlating distance definition for every classifier is marked by a thicker frame around the plot

**Fig. 3:**
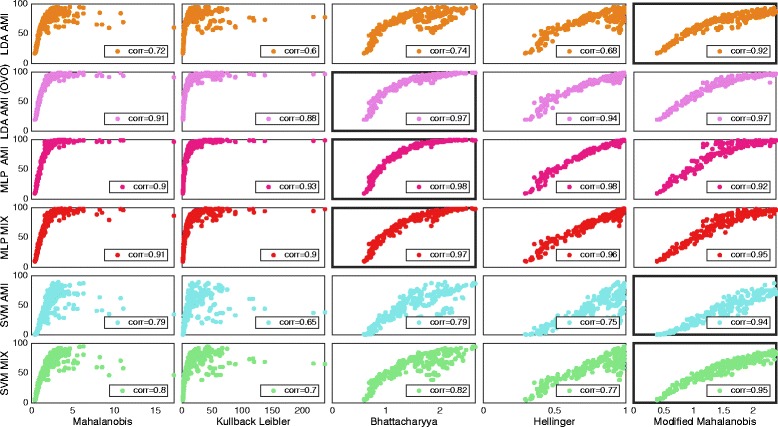
The distribution of distance definitions and classifiers data using simultaneous movements (SM set). Plot matrix where the insets shows classification accuracy plotted against the SI for the simultaneous movements data set. One marker represents the average over all movements for one subject and one feature. The classifiers are grouped in rows and the distance definitions for SI are group in columns. Classifiers were configured using “all movements as individual” (AMI) or “mixed outputs” (MIX). Classifiers were used in the conventional “single” topology, apart from LDA, which was used in “single” and OVO. All correlations were found statistically significant at *p* < 0.01. Classifiers and distance definitions are stated at the left side and the bottom of the plot matrix respectively. The highest correlating distance definition for every classifier is marked by a thicker frame around the plot

The correlations found between classification accuracy and SI using different *distance definitions* are summarized in Table 3, where the highest value for every classifier is highlighted. Figures 2 and 3 shows plots of *average result* for *IM* and *SM data* sets, respectively, with the most correlating *distance definition* highlighted for classifiers individually. Table 3, Figs. 2 and 3 indicates that the most adequate *distance definitions* vary with the classifier.

**Table 3:** Correlations for the different distance definitions

	Average result			Individual results			
	LDA (AMI) Single/OVO	MLP AMI/MIX	SVM AMI/MIX	LDA (AMI) Single/OVO	MLP AMI/MIX	SVM AMI/MIX	Data set
Mahalanobis	0.72/0.91	0.90/0.91	0.79/0.80	0.81/**0.92**	0.84/0.85	0.70/0.68	SM
	0.78/0.88	0.86/NA	0.71/NA	**0.85**/0.91	0.80/NA	0.60/NA	IM
Bhattacharyya	0.74/**0.97**	**0.98**/**0.97**	0.79/0.82	0.69/0.91	0.93/**0.91**	0.66/0.65	SM
	0.83/0.96	0.96/NA	0.68/NA	0.79/0.89	**0.94**/NA	0.68/NA	IM
Kullback–Leibler	0.60/0.88	0.93/0.90	0.65/0.70	0.54/0.76	0.84/0.82	0.63/0.60	SM
	0.51/0.72	0.80/NA	0.32/NA	0.65/0.75	0.87/NA	0.65/NA	IM
Hellinger	0.68/0.94	**0.98**/0.96	0.75/0.77	0.69/0.90	0.93/**0.91**	0.66/0.65	SM
	0.80/0.95	0.97/NA	0.66/NA	0.79/0.89	**0.94**/NA	0.68/NA	IM
Modified Mahalanobis	0.92/**0.97**	0.92/0.95	**0.94**/**0.95**	0.79/0.91	0.88/0.89	**0.74**/**0.71**	SM
	**0.93**/0.94	0.87/NA	0.83/NA	**0.85**/0.90	0.86/NA	0.71/NA	IM

Correlations under “individual results” were calculated using classification accuracies and SIs from every individual movement, subject and feature, while those under “average result” were derived using the average SI and classification accuracy per subject and feature. Both methods provide one correlation, although “individual results” use more data. Classifiers were configured using AMI or MIX. Classifiers were used in the conventional “single” topology, apart from LDA, which was used in “single” and OVO. The highest correlation values per column are highlighted in bold. All correlations were found to be statistically significant at *p* < 0.01. The MIX configuration is not applicable (NA) for individual movements since there is not mixed outputs

#### Mahalanobis distance

**Fig. 4:**
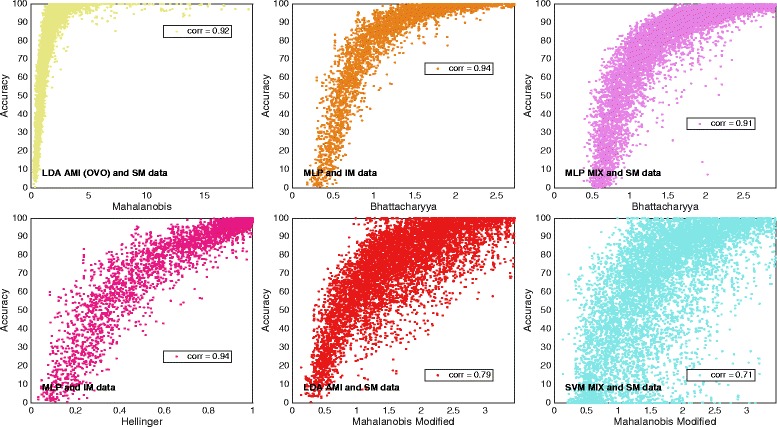
Data distribution for the most correlating distance definitions. Plot matrix where the insets show classification accuracy plotted against the SI. One dot represents one movement, one subject, and one feature, which means that the number of dots is the number of movements multiplied by the number of subjects times the number of features. The plots represent the highlighted correlations in Table 3

*Mahalanobis distance* was found as the *distance definition* that most closely correlated with LDA in an OVO topology for *individual results* using *SM data*. The corresponding classification accuracy against SI is plotted in Fig. 4a.

#### Kullback–Leibler divergence

*Kullback–Leibler divergence* was not found to yield higher correlation than any other *distance definition* for any of the classifiers; however, it was found to correlate most closely with the *average results* of MLP using both topologies. This correlation is visualized in Figs. 2 and 3. Owing to its low correlation with classification accuracy, *Kullback–Leibler divergence* was not used in the reaming experiments.

#### Bhattacharyya distance

*Bhattacharyya distance* was the most correlating *distance definition* for MLP in both *AMI* and *MIX* configurations. Plots of classification accuracy for the two classifiers against SI based on *Bhattacharyya distance* is shown in insets B and C of Fig. 4. *Individual results* are presented and *IM data* and *SM data* are used for *AMI* and *MIX* configurations, respectively.

#### Hellinger distance

*Bhattacharyya distance* and *Hellinger distance* are highly related as they are both based on the *Bhattacharyya Coefficient*. Table 3 confirms their resemblance as the correlations related to the two *distance definitions* are very similar in all cases. Naturally, *Hellinger distance* and *Bhattacharyya distance* are the *distance definitions* that most closely correlate with MLP MIX and AMI for *individual result,* and with MLP *AMI* for *average result*. MLP *AMI* classification accuracy is plotted against *Hellinger distance* based SI in Fig. 4e, where *individual results* using *IM data* is represented.

#### Modified Mahalanobis

*Modified Mahalanobis* was found as the *distance definition* that correlates most closely with *average results* of LDA and SVM classification accuracy for all topologies and configurations. The same is true for *individual results,* except for LDA in an OVO topology*.* Insets E and F of Fig. 4 show LDA *AMI* and SVM *MIX* classification accuracy plotted against SI based on *Modified Mahalanobis. Modified Mahalanobis* was the version of *Mahalanobis distance* used in the remaining results because of its overall higher correlation with classification accuracy.

### Nearest neighbor separabillity (NNS)

A summary of correlations with all classifiers for both data sets is presented in Table 4.

**Table 4:** Correlations between classification accuracy and nearest neighbor separability

	Average result			Individual results			
	LDA (AMI) Single/OVO	MLP AMI/MIX	SVM AMI/MIX	LDA (AMI) Single/OVO	MLP AMI/MIX	SVM AMI/MIX	Data set
K = 20	0.86/**0.98**	0.96/**0.97**	0.90/0.90	0.83/0.93	0.92/0.92	0.72/0.72	SM
	0.86/0.97	0.98/NA	0.74/NA	0.84/0.92	**0.97**/NA	0.70/NA	IM
K = 120	**0.90**/0.97	0.92/0.95	**0.92**/**0.92**	0.87/0.90	0.87/**0.89**	**0.73**/0.73	SM
	**0.90**/**0.98**	**0.97**/NA	0.78/NA	**0.89**/**0.93**	0.94/NA	**0.73**/NA	IM

*The correlation between classification accuracy and NNS with different values of the parameter k. Correlations under “individual results” were calculated using classification accuracies and NNS from every individual movement, subject and feature, while those under “average result” were derived using one average NNS and classification accuracy for every subject and feature. Both methods provide one correlation, although “individual results” use more data. Classifiers were configured using AMI or MIX. Classifiers were used in the conventional “single” topology, apart from LDA, which was used in “single” and OVO. The highest correlation values per column are highlighted in bold. All correlations were found statistically significant at p < 0.01. The MIX configuration is not applicable (NA) for individual movements since there are no mixed outputs*

**Fig. 5:**
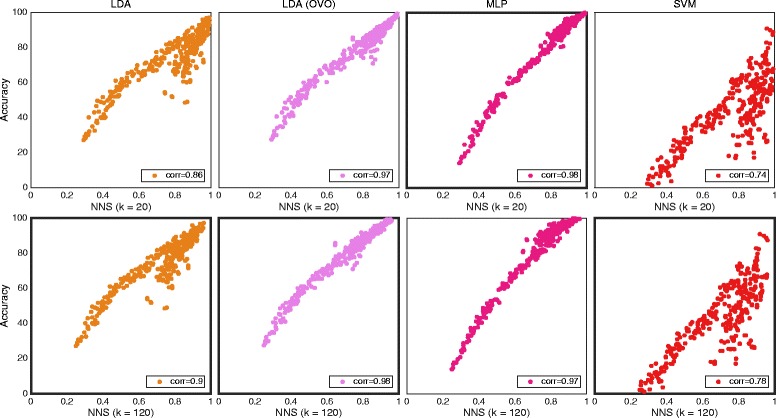
The distribution of data from individual movement for NNS and all classifiers. Plot matrix where the insets shows classification accuracy plotted against NNS for the individual movements data set. One marker represents the average over all movements for one subject and one feature. Classifiers were used in the conventional “single” topology, apart from LDA, which was used in “single” and OVO. All correlations were found statistically significant at *p* < 0.01. The classifiers are grouped in columns and the results for different values of the parameter k are group in rows. The highest correlation values per column are highlighted by a thicker frame

**Fig. 6:**
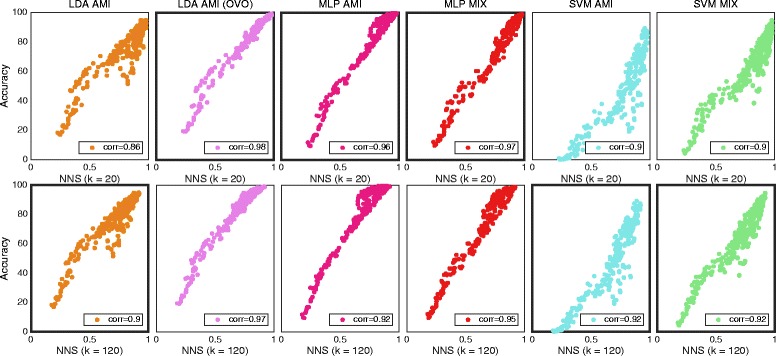
The distribution of data from simultaneous movement for NNS and all classifiers. Plot matrix where the insets shows classification accuracy plotted against NNS for the simultaneous movements data set. One marker represents the average over all movements for one subject and one feature. Classifiers were configured using AMI or MIX. Classifiers were used in the conventional “single” topology, aside of LDA which was used in “single” and OVO. The highest correlation values per column are highlighted by a thicker frame. All correlations were found statistically significant at *p* < 0.01. The classifiers are grouped in columns and the results for different values of the parameter k are group in rows

Table 4 also shows the influence of the parameter *k*. Figures 5 and 6 show plots of *average result* for the *IM* and *SM data*, respectively.

**Fig. 7:**
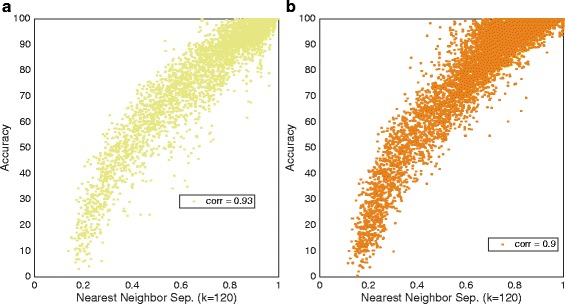
Highest correlation for NNS. LDA (OVO) classification accuracy plotted against NNS for individual result. One dot represents one movements, one subject and one feature, meaning that the number of dots is the number of movements multiplied by the number of subjects multiplied by the number of features. The plots illustrate the highest correlation from Table 4. **a** LDA (OVO) and IM data. **b** LDA AMI (OVO) and SM data

NNS is most correlated with LDA in an OVO topology, which is equivalent to the results obtained by SI based on *Bhattacharyya distance* for the same classifier. The *individual results* for LDA using OVO are plotted for both data sets in Fig. 7.

### Purity and repeatability index

**Fig. 8:**
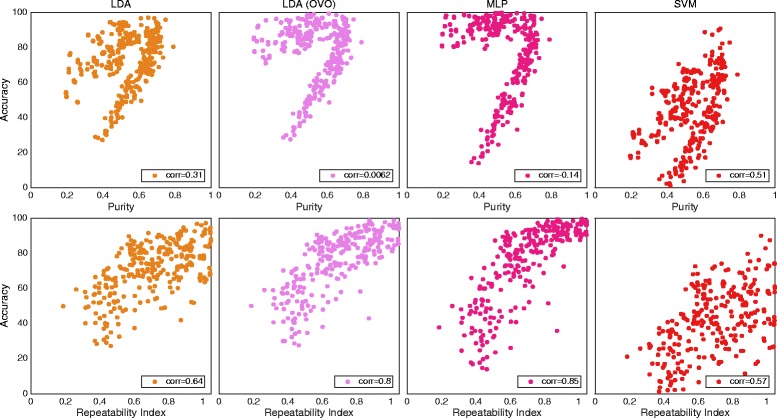
The distribution of data from individual movement for all classifiers with purity and repeatability. Plot matrix where the insets shows classification accuracy plotted against purity for row 1 and repeatability for row 2. The result is for the individual movements data set. One marker represents the average over all movements for one subject and one feature. Classifiers were used in the conventional “single” topology, apart from LDA, which was used in “single” and OVO. The classifiers are grouped in columns

Purity and repeatability index resulted in low correlation with classification accuracy for all classifiers. The correlations for *IM data* can be found in Table 5. Figure 8 shows *Individual results* of MLP for the two algorithms and the aforementioned data set. Because of the low correlation, purity was excluded from the following experiments, and RI from the *Feature Sets* experiment.

**Table 5:** Correlation for purity and repeatability index regarding classification accuracy

	Average result			Individual results			
	LDA (AMI) Single/OVO	MLP AMI	SVM AMI	LDA (AMI) Single/OVO	MLP AMI	SVM AMI	Data set
Purity	0.31/0.0062	−0.14	0.51	0.3/0.15	0.14	0.54	IM
Repeatability	0.64/0.8	0.85	0.57	0.23/0.36	0.45	0.16	IM

*The correlation with classification accuracy for purity and repeatability index. Correlations under “individual results” were calculated using classification accuracies and CCES from every individual movement, subject and feature, while those under “average result” were derived using one average CCE and classification accuracy for every subject and feature. Both methods provide one correlation, although “individual results” use more data. Classifiers were configured using AMI. All correlations were found to be statistically significant (p < 0.05)*

### Feature sets

**Fig. 9:**
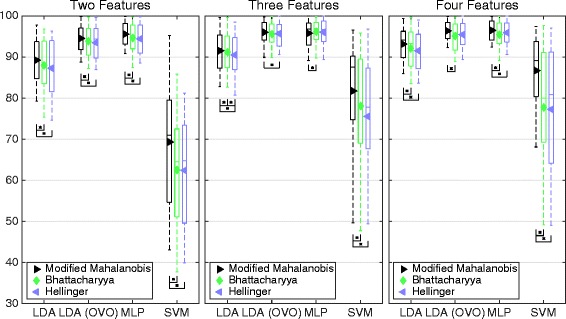
Classification accuracy for the best sets corresponding to distance definitions of SI. Boxplot of average classification accuracy over all movements when using the best sets representing the distance definitions found in the legends. The middle line of the box is the median, the marker is the mean, and the box extends to the 25th and the 75th percentiles for the bottom and the top, respectively. The different insets compare sets of different number of features. The result is derived from the IM data set. Classifiers were used in the conventional “single” topology, apart from LDA, which was used in “single” and OVO

In this section, the *best sets* are compared with each other and the *reference sets.* In Fig. 9, the *best sets* corresponding to the *distance definitions* of SI are compared. The *modified Mahalanobis* sets are significantly higher than the other *distance definitions* sets in eight out of 12 cases, and averagely higher in all but the case where MLP is used with sets of three features. In that case, *Bhattacharyya distance* and *Hellinger distance* sets performing higher average classification accuracy.

**Fig. 10:**
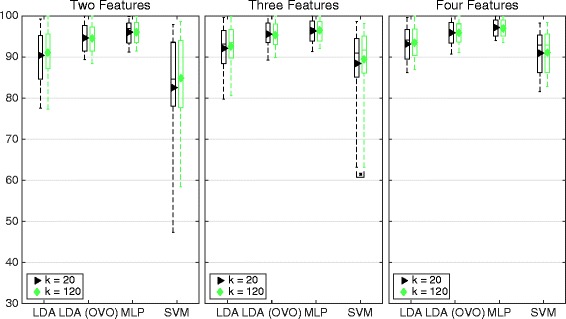
Classification accuracy for the best sets corresponding to distance definitions of the SI. Boxplot of average classification accuracy over all movements when using the best sets representing the distance definitions found in the legends. The middle line of the box is the median, the marker is the mean, and the box extends to the 25th and 75th percentiles for the bottom and the top, respectively. The different insets compare sets of different number of features. The result is derived from the IM data set. Classifiers were used in the conventional “single” topology, apart from LDA, which was used in “single” and OVO

The influence of parameter k of the NNS algorithm is shown in Fig. 10 by comparing the *best sets* for k = 120 and k = 20. The higher value of k leads to higher average classification accuracy in all cases. However, it is statistically significant for SVM and three features only.

**Fig. 11:**
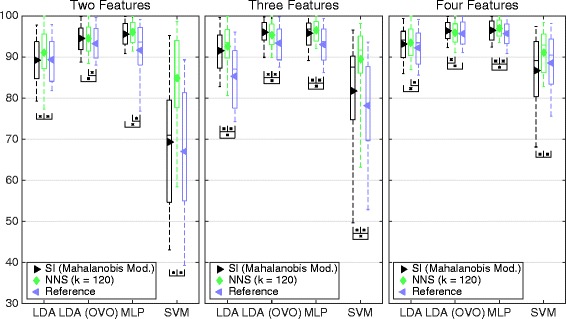
Classification accuracy for the best sets compared to the reference sets. Boxplot of average classification accuracy over all movements when using the best sets representing SI with modified Mahalanobis as distance definition, NNS with k = 120 and the reference sets. The value of k is found in the legend. The middle line of the box is the median, the marker is the mean, and the box extends to the 25th and the 75th percentiles for the bottom and the top, respectively. The different insets compare sets different number of feature algorithms. The result is derived from the IM data set. Classifiers were used in the conventional “single” topology, apart from LDA, which was used in “single” and OVO

The members with the highest average classification accuracy were selected from Figs. 9 and 10 – *modified Mahalanobis* and k = 120, respectively – to be compared with the reference sets in Fig. 11. The NNS sets leads to significantly higher classification accuracy than the reference in all but one case, while *modified Mahalanobis* is significantly higher for nine out of 12. The average classification accuracy for the NNS sets is higher than *modified Mahalanobis* for all classifiers except LDA in an OVO topology, where *Modified Mahalanobis* is consistently higher.

### Real time

**Fig. 12:**
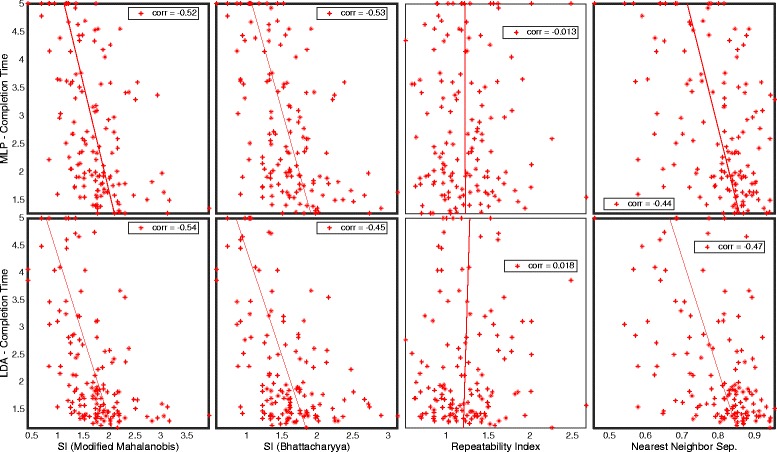
Real-time correlation for Classification Complexity Estimations. Plot matrix where the insets are completion time plotted against classification complexity estimates. Significant correlation (*p* < 0.001) is highlighted with bold frame

Figure 12 summarizes the correlations between the *motion test* result *completion time* and CCEs corresponding to RI, NNS, and SI based on *modified Mahalanobis* and *Bhattacharyya distance*. Statistically significant correlations (*p* < 0.001) are highlighted by a darker frame.

### Feature attribute

**Fig. 13:**
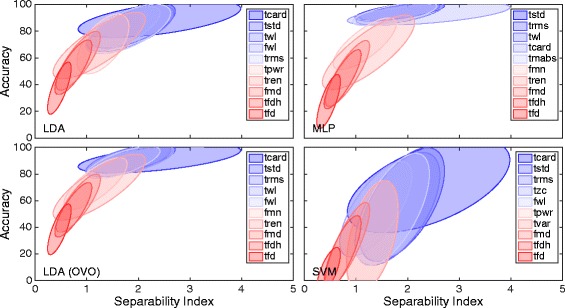
High- and low-performing features for the IM data. Ellipses representing clusters for features in classification accuracy against SI plots for results using the IM data set. The SI distance definition is modified Mahalanobis. The ellipses are centered around the means of the feature clusters and constructed according to their covariance matrices. Every inset includes the features with the top five and bottom five average classification accuracies for the classifier stated in the plot. The ellipses are coded by *red* and *blue* color for low and high average classification accuracy, respectively. Classifiers were used in the conventional “single” topology, apart from LDA, which was used in “single” and OVO

**Fig. 14:**
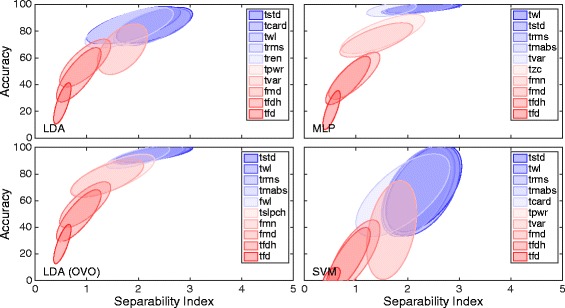
High- and low-performing feature algorithms for the simultaneous movements data**.** Ellipses representing clusters for features in a classification accuracy against SI plots for results using the simultaneous movements data set. The SI distance definition is modified Mahalanobis. The ellipses are centered around the means of the feature clusters and constructed according to the covariance matrix. Every inset includes the feature algorithms with the top five and bottom five average classification accuracies for the classifier stated in the plot. The ellipses are coded by *red* and *blue* color for low and high average classification accuracy, respectively. Classifiers were used in the conventional “single” topology, apart from LDA, which was used in “single” and OVO

As the correlations used to evaluate the CCEAs were derived by use of one feature at a time, attributes of features individually were revealed. Examples of such attributes are average classification accuracy and classification accuracy variance. These two attributes are illustrated in Figs. 13 and 14 for *IM* and *SM data*, respectively. Figure 13 shows the five features that resulted in the highest and lowest average classification accuracy for classifiers separately.

**Fig. 15:**
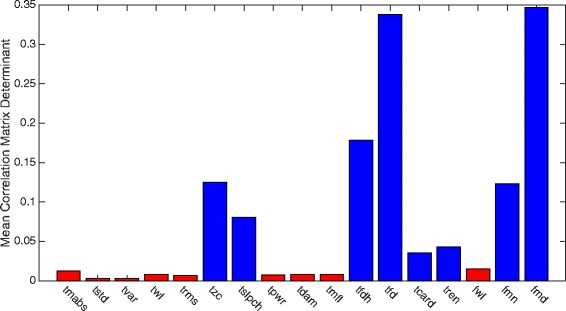
Channel disassociation for features individually. Bar diagram showing the determinant of the correlation matrixes for sets of feature sequences, where one feature was extracted from all channels. The values are averages over all subjects in the simultaneous movement data set. The feature algorithm used is stated at the horizontal axis. A high value means low correlation between channels for the specific feature. The features are divided into two groups depending on their channel correlation. *Red* means high correlation, while *blue* means low

One attribute that was observed to highly influence the CCEAs’ correlation with classification accuracy was channel correlation; that is, correlation between feature sequences extracted from the channels separately using only the feature considered. To illustrate this attribute, average determinants of the channel correlation matrices over all subjects for the different features were extracted from *SM data* and shown in the bar diagram in Fig. 15.

**Fig. 16:**
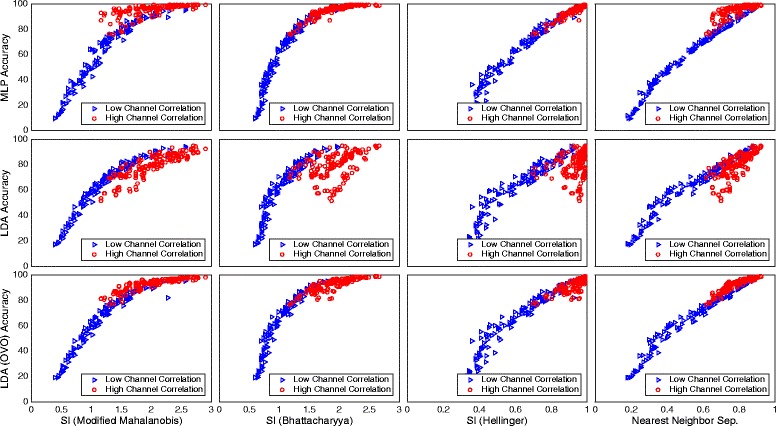
Channel correlation dependency for different classification complexity estimating algorithms. Illustration of how the two feature groups in Fig. 15 cluster in classification accuracy against classification complexity estimate plots. The *red* and *blue* groups result in high and low channel correlation, respectively. Insets in the same row show results from the same CCEA and insets in the same column from the same classifier. Classifiers were used in the conventional “single” topology, apart from LDA, which was used in “single” and OVO

The features marked by red color have low average correlation matrix determinants, which means a high correlation between channels, while the blue color represents features of low channel correlation. Figure 16 shows how the two groups of features, red and blue from Fig. 15, cluster differently in classification accuracy against CCE plots.

The *blue* group has similar dependency on classification accuracy for the three classifiers, while the *red* clearly varies between them.

## Discussion

### Offline results

#### Separability index

*Modified Mahalanobis* was the *distance definition* that had the greatest correlation with classification accuracy (Table 3). However, the *distance definitions* based on *Bhattacharyya coefficient*, being *Bhattacharyya distance* and *Hellinger distance*, had a higher correlation with MLP’s classification accuracy. The *Feature Attributes* section shows that *Bhattacharyya distance* compensates for the change in dependency to MLP classification accuracy caused by input correlation that is found in the other CCEAs. It should therefore be a more adequate *distance definition* for estimation of MLP classification complexity. However, as features are combined into sets, the feature correlation tend to decrease as larger feature vectors are formed using multiple features. This is probably a reason for the absence of significantly higher classification accuracy for *Bhattacharyya distance* (Fig. 9).

#### Nearest neighbor separability

NNS has high correlation with classification accuracy for all classifiers, as shown in Table 4. Figure 10 shows that the *best sets* corresponding to NNS perform higher overall classification accuracy then both the SI *best sets* and the *reference sets.* The greatest benefit of NNS is that it does not assume normality of the distribution, which makes it more general. However, there is a dependency to input correlation, as can be seen in Fig. 16; however, just as for *modified Mahalanobis*, this influence will decrease as features are combined into sets and input correlation decrease.

The drawback of NNS is that it is more computationally demanding than SI. As implemented for this study, the computation time for NNS using two features is approximately 20 and 16 times longer than for SI with *modified Mahalanobis* as *distance definition* using the *IM* and *SM data,* respectively. The absolute time to compute SI in the aforementioned configuration for *IM data* when using Matlab R2015b on a MacBook, 2 GHz Intel Core 2 Duo, 8 GB RAM is approximately 26 ms.

#### Purity and repeatability index

Purity and RI do not show as high correlation with with classification accuracy as the other CCEAs evaluated in this study, and were therefore not included in the feature set experiment. However, the correlation for RI *average result* is relatively high and positive. It is worthy of notice that RI measures the inconsistence during recording. Higher RI means larger cluster shifts in feature space between recording repetitions. Larger shifts were expected to limit the classifiers abilities to identify boundaries and thus reduce classification accuracy.

### Real time

The statistically significant correlations with *completion time* in Fig. 12 argue that both NNS and SI are relevant for prediction of performance in real-time. However, SI with *modified Mahalanobis* as *distance definition* yields higher correlation with *completion time* than NNS, while the offline tests show that the NNS *best sets* are performing with higher classification accuracy for both MLP and LDA also represented in the real-time test. The parametric models of the distributions used for SI are probably more robust to changes present in a real-time situation, similar to what is shown for LDA, also dependent on the assumption of normality [23].

We expected consistent intra-class distribution in feature space, as represented by RI, to be beneficial in the real-time tests, but the low correlation with *completion time* in Fig. 12 does not confirm that hypothesis.

**Fig. 17:**
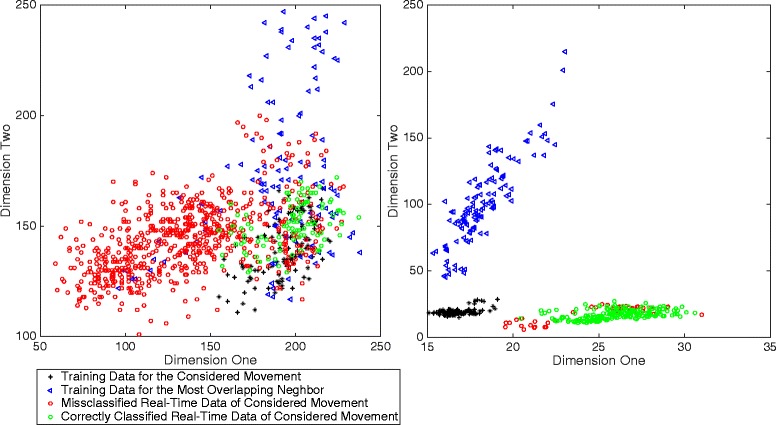
Real-time classification complexity illustration. Scatter plots of the classifier training data, together with the corresponding real-time test data. The inset to the left represents the movement with the highest average completion time of the subject with the overall highest average completion time. The inset to the right represents the movement with the lowest average completion time of the subject with the overall lowest average completion time. The plots also include the movement with the lowest modified Mahalanobis to the movement being considered. The two dimensions that are used to plot the data were selected from the 16 dimensions of the classification task so that the modified Mahalanobis between the training data and the real-time data was maximized

Even though correlations between the CCEAs and the *completion time* are significant for many CCEAs, the correlations with offline accuracy are clearly higher. The complexity of real-time testing is illustrated in Fig. 17, where classifier training data is compared to corresponding real-time data for one movement per inset.

**Fig. 18:**
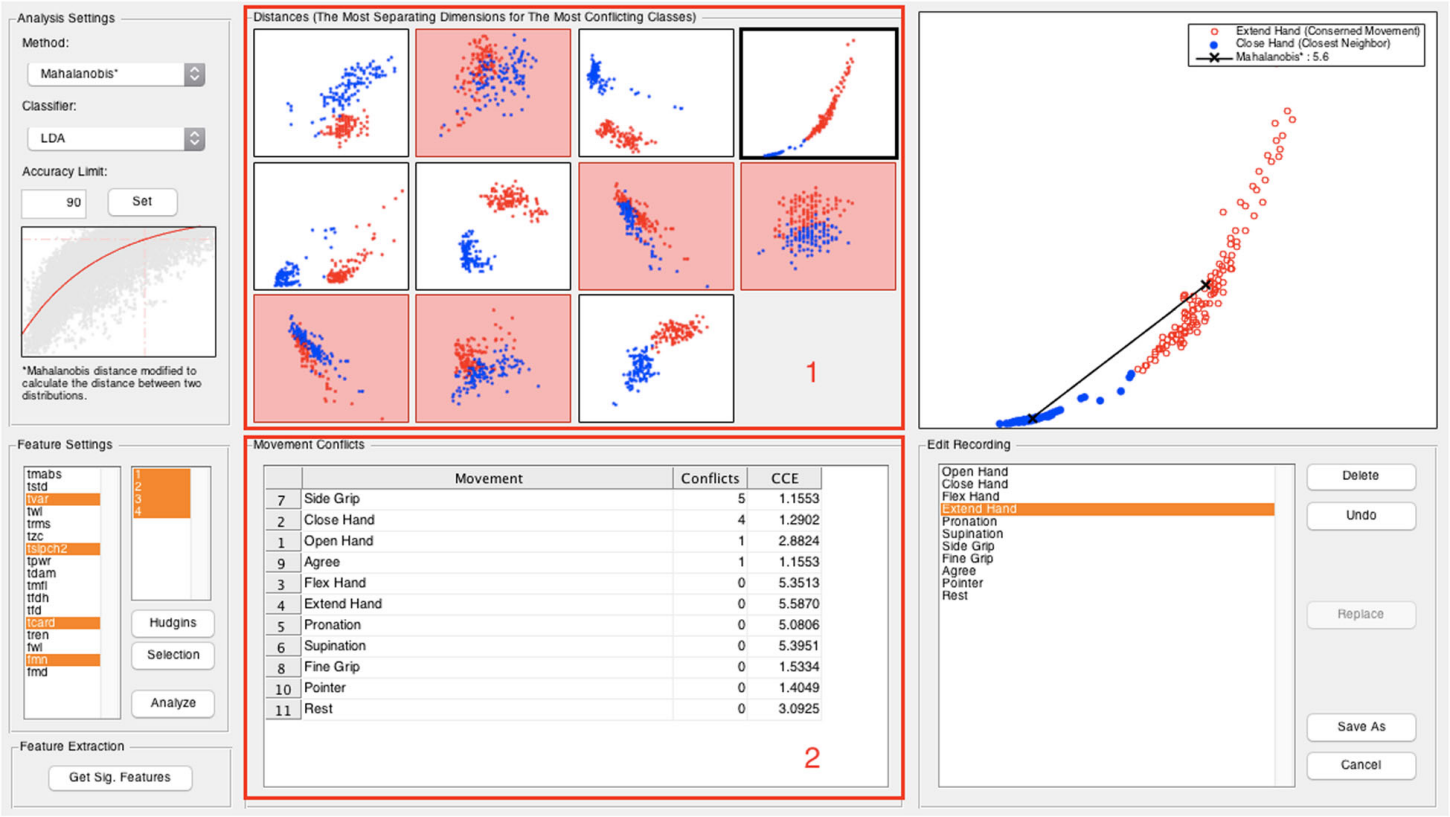
Data analysis tool using CCEAs. Data analysis module integrated into the open-source project BioPatRec [15]. A demonstration case shows data from a recording session of 11 classes (movements). Section 1 shows the feature space per pair of features (2D) for each class and closest neighbors. Section 2 shows the number of times that a movement is the most conflicting neighbor for any of the other movements. The displayed information is derived from the SI (either Bhattacharyya distance or the modified Mahalanobis as distance definitions), or by NNS. This tool provides useful information to identify classes that should be re-recorded or eliminated in order to improve the separability of the others

The distribution clearly shifts between the time when training data was recorded and the time when the real-time test was executed.

### Channel correlation dependency and feature attributes

The change in dependency between CCEs and classification accuracy due to channel correlation of the features presented in the Channel Correlation Dependency section reveals some interesting attributes of the classifiers. Figure 16 shows that features with high channel correlation result in higher average classification accuracy for MLP compared to LDA, but LDA used in an OVO topology is less influenced by the feature correlation. MLP uses the redundant information in the features more efficiently than what is observed for LDA, which suggests that redundancy reduction is of higher importance when selecting both channels and features for a LDA application.

The feature attributes emphasized in Figs. 13 and 14 provide information about the performance of the features in different setups. The variation in the top five features shows how dependent the features’ performance is to other conditions of the classification task, which emphasizes the importance of dynamic feature selection methods for MPR.

### Data analysis tool: example

We implemented the best-performing CCEAs found in this work in a new module for data analysis in BioPatRec [15]; namely, Separability Index with both Bhattacharyya distance and Modified Mahalanobis, and the Nearest Neighbor Separability. The graphical user interface of this module is shown in Fig. 18. Scatter plots show the feature space of different movements and their neighbors. Information about the most conflicting classes based on their interference with other movements is displayed in table format. These attributes are derived from the selected algorithm and are useful inputs when deciding whether to re-record or exclude a particular movement(s).

## Conclusion

This study compared algorithms that estimates the classification complexity of MPR. Two such algorithms, Separability Index (SI) and Nearest Neighbors Separability (NNS), were found to yield high correlation with classification accuracy. The utility of these algorithms for MPR was demonstrated with the high classification accuracy yielded by the feature sets selected using these two algorithms. SI was evaluated using different *distance definitions,* from which best performance was achieved using a modified version of the *Mahalanobis distance,* which also considers the covariance of the neighboring class*.* Overall, the offline results indicated that NNS is a more stable CCEA, while SI is less demanding to compute. In addition, feature correlation dependency was found to influence the correlation between CCEs and classification accuracy.

## Abbreviations

ADC: Analog-to-digital converter
AMI: All movements as individual
CCE: Classification complexity estimates
CCEA: Classification complexity estimating algorithm
EMG: Electromyography
IM: Individual movements
LDA: Linear discriminant analysis
MLP: Multi-layer perceptron
MPR: Myoelectric pattern recognition
NNS: Nearest neighbor separability
OVO: One vs. one
RI: Repeatability index
SI: Separability index
SM: Simultaneous movements
SVM: Support vector machine
